# Defective iron homeostasis and hematological abnormalities in Niemann-Pick disease type C1

**DOI:** 10.12688/wellcomeopenres.17261.2

**Published:** 2023-04-03

**Authors:** Oscar C W Chen, Stephan Siebel, Alexandria Colaco, Elena-Raluca Nicoli, Nick Platt, Dawn Shepherd, Stephanie Newman, Andrew E Armitage, Nicole Y Farhat, George Seligmann, Claire Smith, David A Smith, Alaa Abdul-Sada, Mylvaganam Jeyakumar, Hal Drakesmith, Forbes D Porter, Frances M Platt

**Affiliations:** 1 Department of Pharmacology, University of Oxford, Mansfield Road, Oxford, Oxfordshire, OX1 3QT, UK; 2 Division of Translational Medicine, Eunice Kennedy Shriver National Institute of Child Health and Human Development, National Institutes of Health, Department of Health and Human Services, Bethesda, MD, 20892, USA; 3 MRC Human Immunology Unit, Weatherall Institute of Molecular Medicine, University of Oxford, Oxford, Oxfordshire, OX3 9DS, UK; 4 Chemistry Department, School of Life Sciences, University of Sussex, Brighton, Sussex, BN1 9QJ, UK

**Keywords:** Niemann-Pick disease type C, iron, haematology, lysosome, lysosomal storage diseases

## Abstract

**Background**: Niemann-Pick disease type C1 (NPC1) is a neurodegenerative lysosomal storage disorder characterized by the accumulation of multiple lipids in the late endosome/lysosomal system and reduced acidic store calcium. The lysosomal system regulates key aspects of iron homeostasis, which prompted us to investigate whether there are hematological abnormalities and iron metabolism defects in NPC1.

**Methods**: Iron-related hematological parameters, systemic and tissue metal ion and relevant hormonal and proteins levels, expression of specific pro-inflammatory mediators and erythrophagocytosis were evaluated in an authentic mouse model and in a large cohort of NPC patients.

**Results**: Significant changes in mean corpuscular volume and corpuscular hemoglobin were detected in *Npc1* ^-/-^ mice from an early age. Hematocrit, red cell distribution width and hemoglobin changes were observed in late-stage disease animals. Systemic iron deficiency, increased circulating hepcidin, decreased ferritin and abnormal pro-inflammatory cytokine levels were also found. Furthermore, there is evidence of defective erythrophagocytosis in *Npc1* ^-/-^ mice and in an *in vitro *NPC1 cellular model. Comparable hematological changes, including low normal serum iron and transferrin saturation and low cerebrospinal fluid ferritin were confirmed in NPC1 patients.

**Conclusions**: These data suggest loss of iron homeostasis and hematological abnormalities in NPC1 may contribute to the pathophysiology of this disease.

## Abbreviations

Niemann-Pick disease type C1 (NPC1), hematocrit (HCT), mean corpuscular volume (MCV), hemoglobin (HGB), mean corpuscular hemoglobin (MCH), mean corpuscular hemoglobin concentration (MCHC), red blood cell distribution width (RDW), transferrin (Tf), transferrin receptor (Tfrc), soluble-form of transferrin receptor (sTfrc), soluble transferrin receptor (sTfR), light chain ferritin (L-ferritin), ferroportin (Fpn), interleukin-1 beta (IL-1β), interleukin-1 alpha (IL-1α), tumor necrosis factor alpha (TNFα), hypoxanthine-guanine phosphoribosyltransferase (HPRT) cerebrospinal fluid (CSF), resistance-nodulation-cell division (RND), annual severity increment score (ASIS), endoplasmic reticulum (ER)

## Introduction

Iron is an essential element required as a cofactor for many metalloproteins including hemoglobin (HGB), myoglobin and cytochromes. Systemic iron homeostasis is achieved by controlling the level of circulating iron via its deposition and release from hepatic stores so as to prevent detrimental iron deficiency or excess. It is also influenced by the demand for erythropoiesis and conditions of inflammation and infection ^1^. Disruption of systemic iron homeostasis impairs erythropoiesis and systemic oxygen utilization ^2^; therefore, mammals have evolved complex absorption, recycling, distribution and storage mechanisms to regulate systemic iron metabolism ^3^. 

The lysosome degrades and recycles macromolecules, including iron regulators, transporters and storage proteins ^4– 7^. Cellular iron release from transferrin (Tf) endocytosis, as well as that derived from ferritinophagy, the autophagic process through which intracellular iron is increased via lysosomal degradation of ferritin ^5, 8^ and mitophagy, the selective degradation of damaged mitochondria ^9^ takes place in the endosome/lysosome system ^10^. Iron is subsequently released from the lysosome and distributes it to other subcellular organelles, e.g., mitochondria ^5, 11– 15^. Erythrophagocytosis recycles iron from heme derived from senescent red cells that are ingested by tissue macrophages and delivered into the phago-lysosomal pathway ^16^. 

Niemann-Pick disease type C1 (NPC1) is a lysosomal storage disorder, caused by mutations in either *NPC1* (95% of cases) or *NPC2* and occurs at a frequency of approximately 1:120,000 live births ^17^. The exact biological functions and molecular interactions of the NPC1 and NPC2 proteins remain elusive ^18, 19^, however, the disease is characterized by reduced calcium ion content of the lysosome (acidic store calcium) and accumulation of un-esterified cholesterol and sphingolipids in the late endosomal/lysosomal system ^20, 21^ that result in part from failure in lysosome: endoplasmic reticulum (ER) contact site formation ^22^. NPC1 typically presents as a progressive neurodegenerative disease of infancy/childhood, but adulthood onset forms have been described ^17^.

In the current study, we investigated hematological changes and iron metabolism in an authentic murine model of NPC1 ( *Npc1 ^-/-^*) and in NPC1 patients. We found low serum iron, HGB and mean corpuscular HGB (MCH) in *Npc1* ^-/-^ mice and low iron and Tf saturation levels in NPC1 patients. Furthermore, the decreased systemic iron in *Npc1* ^-/-^ mice correlates with systemic inflammation, significantly increased circulating hepcidin levels and impaired phagocytic clearance of erythrocytes. Comparable hematological changes and cerebrospinal fluid (CSF) ferritin deficiency were detected in NPC1 patients. These studies suggest that loss of systemic iron homeostasis induces hematological changes in NPC1 via multiple mechanisms and that NPC1 patients may be at risk of iron deficiency.

## Methods

### Reagents

Reagents were from Sigma-Aldrich unless otherwise specified.

### Animals

Niemann-Pick disease type C1 mice (BALB/cNctr- *Npc1m1N*/J; *Npc1* ^-/-^) ^23^ were housed at the University of Oxford. Food and water were available *ad lib*. Iron content in the diet was 200 mg/kg (Teklad Global 16% protein rodent diet, Harlan Laboratories). Animal studies were authorized by the UK Home Office (Animal Scientific Procedures Act, 1986). *Npc1* ^-/-^ mice have a lifespan of 10–12 weeks (average 10.5 weeks) with neurological symptoms presenting from seven weeks of age. Mice were sampled at multiple ages; early pre-symptomatic (three-weeks-old), pre-symptomatic (five-weeks-old), early-symptomatic (seven-weeks-old), late-symptomatic (nine-weeks-old) stage and late end stage (eleven-weeks-old). Male animals were used in all studies, except where indicated.

All experiments involving animals were conducted under the authority of project licence number PPL P8088558D, approved by the University of Oxford Animal Welfare and Ethical Review Body and granted by the United Kingdom Home Office (Animal Scientific Procedures Act, 1986). Animals were housed in the Biomedical Research Services facilities, University of Oxford. All licensed procedures were performed in accordance with the United Kingdom Animals (Scientific Procedures) Act 1986. All efforts were made to ameliorate any suffering of animals including adapting water and food provision during the symptomatic phase of the Npc1 deficient mice used in this study. The study design for each investigation involving the use of animals or animal-derived tissues adhered to criteria designated by the ARRIVE Essential Checklist. Mice were generated from in-house breeding colonies, genotyped, sex and age matched and allocated randomly for investigation. Where possible, mice of the two genotypes were co-housed.

### Mouse hematological analysis

Mice were sacrificed at the indicated ages using approved Schedule 1 methods including overdose of anaesthetic. Blood (volumes < 2ml) was collected by cardiac puncture. Multiple hematological parameters were determined using a Pentra ES 60 system blood analyzer (HORIBA-ABX). Blood samples were collected from mice and analyzed immediately. Blood smears were stained with Wright-Giemsa reagent, examined on a Zeiss Axioplan 2 microscope and images captured using Axiovision 2.0 software.

### Mouse serum preparation and iron tissue determinations

Blood samples were collected and allowed to clot, spun at 3,000 rpm for 15 minutes and serum stored at -20°C.****** Sera were diluted 200-fold with 1% nitric acid at 70°C overnight. Mouse tissues were flash frozen and digested in nitric acid (69%). Samples were diluted 50-fold in water (VWR, 83877.290). Total elemental iron was measured by inductively coupled plasma mass spectrometry (ICP-MS) as previously described ^24^. In brief, tissues were heat-digested completely in nitric acid and metal content quantified on a Thermo Finnigan Element 2 Sector-Field ICP-MS. Rhodium (1ng/g) was spiked into each sample as an internal standard. Iron concentrations were normalized to starting tissue weight.

### Hepcidin measurements

Blood samples were collected, serum prepared and hepcidin levels were determined using the Hepcidin Murine-Compete ELISA kit according to the manufacturer’s instructions (Intrinsic Life Sciences).

### Western blotting

Tissues or serum were collected, and lysates prepared by homogenization in cell lysis buffer (Cell Signaling Technology) containing protease inhibitors (cOmplete EDTA-free protease inhibitor cocktail, Merck) on ice, followed by centrifugation to remove insoluble protein. Protein concentration of supernatants was determined by BCA assay (Sigma). Appropriate volumes of lysates were mixed with SDS Blue loading buffer (BioLabs) and heated to 95°C for 5 min and then rapidly cooled on ice. Samples were loaded onto NuPAGE ^TM^ Bis-Tris Gels (ThermoFisher) and run in NuPAGE ^TM^ MOPS SDS running buffer (ThermoFisher). Novex Sharp Pre-stained protein standard (ThermoFisher) was used to indicate the extent of protein migration and specific protein mass. Gels were transferred onto Immuno-Blot ^®^ PVDF membrane (Bio-Rad) using BIORAD transblot turbo transfer system (Bio-Rad). Membranes were blocked with 5% skimmed milk in PBS with 0.1% Tween 20 (Sigma) for 1h at room temperature and then incubated with primary antibody (rabbit polyclonal anti-mouse light chain ferritin (L-ferritin) antibody was from Abcam (ab69090), used at 1: 1000 **;**rabbit anti-mouse soluble transferrin receptor (sTfrc) polyclonal antibody was from Fisherscientific (17278842), used at 1: 500, diluted in PBS containing 2.5% skimmed milk with 0.1% Tween 20 and sodium azide overnight at 4°C. Membranes were washed with PBS containing 0.1% Tween 20 three times for 20 min each and then incubated with HRP-conjugated donkey anti-rabbit polyclonal antibody from Jackson Immunochemicals (711-035-152), diluted to 1:5000 in PBS containing 2.5% skimmed milk and 0.1% Tween 20 for 1h at room temperature. Membranes were washed as before and then developed with SuperSignal ^TM^ West Femto substrate (ThermoFisher) or Pierce ^TM^ ECL western blotting substrate (ThermoFisher). Membranes were re-probed with anti-b-actin antisera (Sigma) to evaluate equivalent protein loading. Images were obtained using a ChemiDoc XRS system (Bio-Rad) and processed and analyzed with ImageLab 5.1 software (Bio-Rad) with BioRad Universal Hood.

### Q-PCR

Mouse tissues were snap frozen in liquid nitrogen. RNA was isolated using RNeasy kits (Qiagen) according to manufacturer’s protocol and quantified using Nanodrop spectrophotometer (Thermo Scientific), cDNA generated with iScript cDNA synthesis reagents (Bio-Rad) and duplicate qPCR reactions set up with 5ng template cDNA/RNA, PowerUP SYBR Green Master Mix (Thermo Fisher Scientific) and specific primers ( Table 1). Reactions were run on CFX96 Real-Time PCR Detection System (Bio-Rad) with amplification of HPRT as internal housekeeping control. Cycling conditions were: UDG activation, 50°C for 2 min; Dual-Lock DNA polymerase, 95°C for 2 min and 40 cycles of denaturation at 95°C for 15 sec and anneal/extend at 60°C for 30 sec. Gene expression levels were calculated from Ct values using comparative Ct methodology and plotted as relative to the HPRT control.

**Table 1.** Details of primers used for Q-PCR analysis.

Name	Sequence	Product size
mTfrc set2F	TCCGCTCGTGGAGACTACTT	140bp
mTfrc set2R	ACATAGGGCGACAGGAAGTG	
mFerroportin F	TTGCAGGAGTCATTGCTGCTA	161bp
mFerroportin R	TGGAGTTCTGCACACCATTGAT	
mTNFalpha F	CCCTCACACTCAGATCATCTTCT	61bp
mTNFalpha R	GCTACGACGTGGGCTACAG	
mIL-1alpha F	CGCTTGAGTCGGCAAAGAAA	107bp
mIL-1alpha	AGATGGTCAATGGCAGAACTGT	
mIL1beta F	AAGGAGAACCAAGCAACGACAAAA	213bp
mIL1beta R	TGGGGAACTCTGCAGACTCAAACT	
mHPRT F	CAAACTTTGCTTTCCCTGGT	101bp
mHPRT R	TCTGGCCTGTATCCAACACTTC	
mACTB F	GGCTGTATTCCCCTCCATCG	154bp
mACTB R	CCAGTTGGTAACAATGCCATGT	
NPC1wt F	TCTACGCTGATTACCACACACA	112bp
NPC1wt R	AACACCGGTCCTCCAAATGT	

### Histology and immunohistochemistry

Mice were sacrificed by Schedule 1 protocol, perfused with 4% paraformaldehyde, tissues removed and embedded and 4 μm paraffin sections stained with hematoxylin/eosin and Masson trichrome images were collected on a Zeiss Axioskop 2 microscope using Axiovision 2 software. 

### Flow cytometry

Single-cell spleen suspensions from nine-week-old mice were prepared by physical disruption and passage through single cell strainers (Fisher) and stained with PE-conjugated anti-mouse TER-119 (BD Bioscience, 553673; used at 5mg/ml) and FITC-conjugated anti-mouse CD71 antibodies (BioLegend, 113805, used at 10mg/ml) and labelled using LIVE/DEAD Cell Viability Assay Kit (ThermoFisher L34955). Live cell data were acquired on a FACSCanto II Flow Cytometer (BD Biosciences) and analysed using FlowJo 10.2 software, LLC. 

### *In vitro* Ox-RBC phagocytosis assay

RAW 264.7 MΦ were obtained from ATCC and maintained in RPMI 1640 (Sigma) containing 10% (v/v) foetal bovine serum, 1% penicillin-streptomycin and 1% L-glutamine. Cells were passaged at regular intervals to maintain viability > 90%. Cells were treated with vehicle (DMSO) or 2 μg/ml U18666A (Merck) for 24 h prior to erythrophagocytosis assay. Sheep red blood cells (TCS Biochemicals) were labelled with CellTracker Green CMFDA Dye (Invitrogen), incubated with 0.2 mM CuSO _4_ and 5 mM ascorbic acid for 1h ^25^, overlaid onto MΦ plated onto glass coverslips and co-incubated at 37°C. At the times indicated, non-ingested erythrocytes were removed by washing, phagocytes fixed and examined by confocal microscopy (Leica TPC SP8) running Leica Application Suite software (LAS X) and images analyzed using ImageJ Fuji (NIH, USA). Ingestion by a minimum of 300 MΦ was determined for each condition.

### Patient sample collection

NPC1 patients were enrolled in a longitudinal observational study at the National Institutes of Health, Bethesda, USA approved by the NICHD Institutional Review Board (06-CH-0186). Written informed consent, and assent was obtained as appropriate. Diagnosis was established by biochemical testing/mutation analysis. Phenotypic severity was determined using the annualized severity increment score developed by Yanjanin *et al.* ^26^, that measures symptoms in nine major and eight minor clinical areas, which are primarily neurological. Scores ranged from one to 35 (max severity on this scale is 50). Serum samples were excluded from this study if they were collected from patients with a history of splenectomy (n=2) or thalassemia (n=1). Blood was analyzed immediately at the NIH Clinical Center Department of Laboratory Medicine (DLM). Iron, transferrin and percent saturation were measured using the Dimension Vista ^®^ System 1500 at the NIH DLM. Data from only one sampling time point was used for each individual participant. CSF samples were obtained by lumbar puncture within the L4/L5 interspace. CSF was stored at -80°C prior to assay by Medical Neurogenetics, LLC. STROBE reporting guidelines were adhered to in the observational studies.

### Patient soluble-transferrin receptor, C-reactive protein and ferritin analysis

Patient blood samples were collected and processed to obtain serum. sTfR was measured using Quantikine ELISA Kit (R&D Systems) according to the manufacturer’s protocol. Plasma C-reactive protein (CRP, MULTIGENT CRP Vario Kit, with high sensitivity calibrators) and ferritin (Architect Ferritin Assay) were analyzed using the Abbott Architect 2000R automated analyzer (Abbott Laboratories) at Birmingham Heartlands hospital (Birmingham, UK).

### Enzyme linked immunosorbent assay (ELISA) for human TNF-α

Serum TNF-α levels of 22 patients and 14 control individuals of comparable age and gender distribution were measured by ELISA (Thermo Scientific) according to the manufacturer’s instructions.

### CSF ferritin analysis

CSF was collected from patients as part of their clinical evaluation. CSF ferritin was measured using a human ferritin ELISA kit (Abnova) according to the manufacturer’s instructions.

### Statistical analysis

Data are expressed as mean ± SEM. Statistical analysis was performed using GraphPad Prism 9 (Dotmatrics). Unpaired two-tailed Student’s *t* test or ANOVA were used to determine significance. *p* < 0.05 were considered significant. * *p* < 0.05, ** *p* < 0.01, *** *p* < 0.001, **** *p* < 0.0001.

## Results

### Altered erythrocyte parameters and erythrocyte morphology in *Npc1 ^-/-^* mice

**Figure 1.**
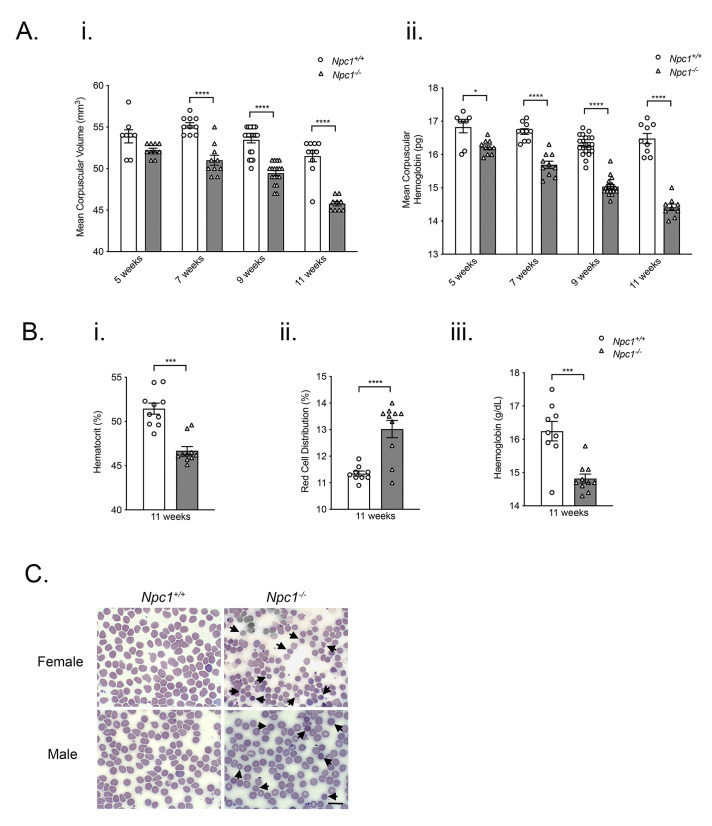
Altered erythrocytic indices and erythrocyte morphology in *Npc1 ^-/-^* mice. **A**. Histograms showing significantly lower mean corpuscular volume ( **i**) and mean corpuscular hemoglobin ( **ii**) in five-week, seven-week, nine-week and eleven-week-old *Npc1 ^-/-^* mice. **B**. Histograms representing reduced hematocrit ( **i**), increased red cell distribution width ( **ii**) and decreased hemoglobin ( **iii**) in eleven-week *Npc1 ^-/-^* mice. Data shown are mean ± SEM, n=7–20 mice per group. * *p* < 0.05 ** *p* < 0.01*** *p* < 0.001, **** *p* < 0.0001. 1-way ANOVA with Tukey’s. **C**. Wright-Giemsa stain of peripheral blood smears from nine-week-old male and female *Npc1 ^+/+^* and *Npc1 ^-/-^* mice. Arrowheads indicate examples of erythrocytes with altered morphology. Scale bar represents 10 μm. Images of blood smears were taken on Zeiss Axioplan 2 microscope and captured using Axiovision 2.0 software. Data are representative of three independent experiments.

To investigate the impact of lysosomal dysfunction on erythropoiesis, we measured multiple erythrocyte-related parameters in *Npc1 ^-/-^* mice at different stages of disease progression (five weeks, pre-symptomatic; seven weeks, early symptomatic; nine weeks, late symptomatic and eleven weeks, end stage). Mean corpuscular volume was significantly decreased in *Npc1* ^-/-^ mice at seven-weeks-of-age and remained lower at all later time points ( *p* < 0.0001; n=10–20) ^27^ ( Figure 1Ai). Corpuscular hemoglobin (MCH) was significantly lower from five-weeks of age and was decreased at all subsequent ages ( *p* < 0.0001; n=10–18) ( Figure 1Aii). Hematocrit (HCT) was significantly reduced at eleven-weeks-of-age ( *p* < 0.0001; n=10) ( Figure 1Bi); red cell distribution width (RDW) was increased ( *p* < 0.0001; n=9–10) ( Figure 1Bii) and HGB reduced ( *p* < 0.0001; n=10) ( Figure 1Biii) but were not significantly different at earlier ages. Mean corpuscular hemoglobin concentration (MCHC) and red blood cell number were broadly unchanged at all time points (Extended Figure 1). Wright-Giemsa staining revealed that blood from nine-week *Npc1 ^-/-^* mice contained erythrocytes which were microcytic and irregularly shaped ( Figure 1C).

### Decreased serum iron and increased sTfc in *Npc1 ^-/-^* mice

**Figure 2.**
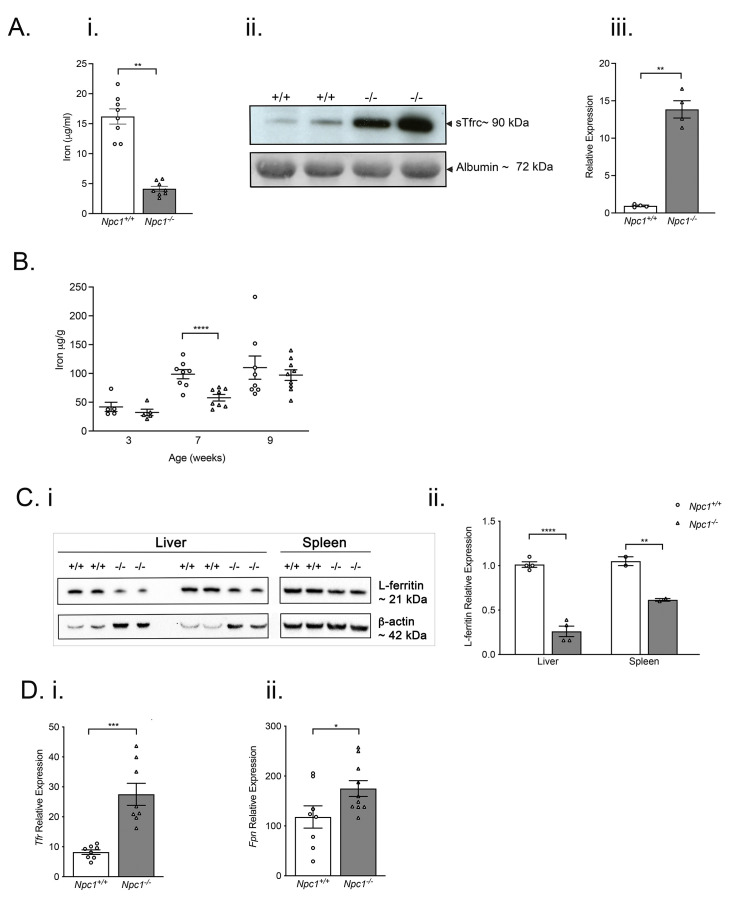
Decreased systemic iron, increased serum sTfrc, transiently reduced hepatic iron and lower L-ferritin and enhanced *tfrc* and *fpn* expression in *Npc1 ^-/-^* mice. Serum iron levels are significantly lower in nine-week-old *Npc1 ^-/-^* mice than in age-matched control animals ( **i**) whereas serum sTfrc are enhanced. Iron data are mean ± SEM, * *p* < 0.05, ** *p* < 0.01 n=8 per group, unpaired *t* test with Welch’s correction. Data are representative of two independent experiments. Western blot of serum protein samples, ( **ii**) quantification of specific protein bands. Data are mean ± SEM, ** *p* < 0.01 n=4 per group, unpaired *t* test with Welch’s correction. ( **iii**). **B**. Hepatic iron is significantly reduced in seven-week-old *Npc1 ^-/-^* mice (filled circles) but not different from controls (filled triangles) at three and eleven weeks of age. Data are mean ± SEM, **** *p* < 0.0001 n= 5–8 per group, 2-way ANOVA ( **i**). **C**. Reduced ferritin content of liver and spleen in nine-week-old *Npc1 ^-/-^* mice. Western blot of liver and spleen lysates ( **i**) quantification of specific bands ( **ii**) Data are mean ± SEM, ** *p* < 0.01 **** *p* < 0.0001 n=2–4 per group, unpaired *t* test with Welch’s correction. Data are representative of two independent experiments. Arrowheads indicate molecular mass of specific proteins. **D**. Increased hepatic *Tfrc* ( **i**) and duodenal *fpn* ( **ii**) transcripts in symptomatic *Npc1 ^-/-^* mice (filled columns) as compared to *Npc1 ^+/+^* mice (open columns). Data are mean ± SEM, * *p* < 0.05 *** *p* < 0.001n=8 per group. Data are representative of three independent experiments.

Compared with age-matched control littermates, nine-week-old *Npc1* ^-/-^ mice had significantly lower serum iron ( *p* < 0.05; n=5) ( Figure 2Ai). Serum sTfR was significantly increased (approximately 12-fold) in nine-week-old *Npc1* ^-/-^ mice ( Figures 2Aii and iii). 

### Reduced hepatic L-ferritin, increased hepatic transferrin receptor (Tfrc) and increased duodenal ferroportin (fpn) expression

To better understand tissue iron homeostasis, we quantified hepatic iron, L-ferritin and *Tfrc* and duodenal *fpn* expression. Although hepatic iron was significantly reduced in seven-week-old *Npc1 ^-/-^* mice ( *p* < 0.01; n=3), there was no significant difference between genotypes in older mice ( Figure 2Bi). Brain iron was not significantly changed (Extended Figure 2). L-ferritin content of liver and spleen was significantly reduced in nine-week-old *Npc1 ^-/-^* mice ( Figures 2Ci and ii). Hepatic expression of *Tfrc* mRNA was significantly higher ( *p* < 0.05; n=8) ( Figure 2Di) as was *fpn* in the duodenum ( *p* < 0.05; n=8) ( Figure 2Dii). There was no significant difference in the iron content of brains taken from *Npc1 ^+/+^* and *Npc1 ^-/-^* mice at all time points examined (Extended Figure 2).

### Significantly increased systemic hepcidin and hepatic pro-inflammatory cytokines in *Npc1 ^-/-^* mice

**Figure 3.**
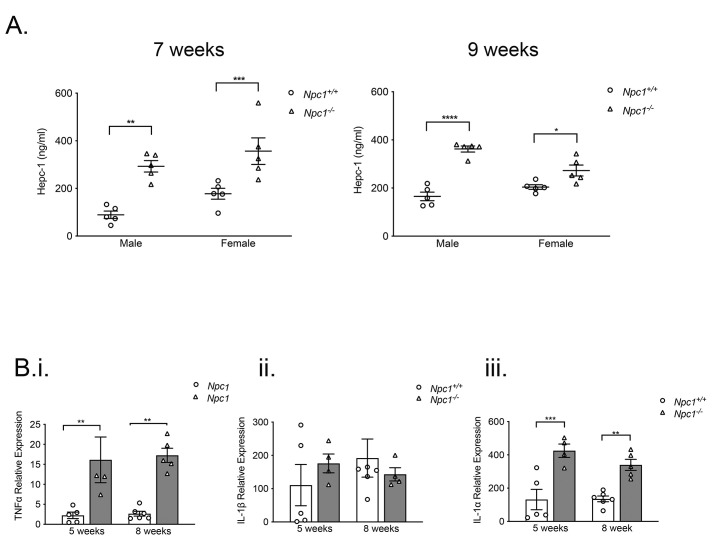
Significantly increased systemic hepcidin and hepatic pro-inflammatory cytokines in *Npc1 ^-/-^* mice. **A**. Male and female *Npc1 ^-/-^* mice (filled circles) have higher systemic hepcidin at seven weeks of age (left panel) and nine weeks of age (right panel) than *Npc1 ^+/+^* mice (filled triangles). Data shown mean ± SEM, * *p* < 0.05, ** *p* < 0.01 *** *p* < 0.001 **** *p* < 0.0001 n=5 per group. 2-way ANOVA. **B**. Q-PCR data confirming ( **i**) increased hepatic TNFα transcripts, ( **ii**) unchanged IL-1β and ( **iii**) enhanced IL-1α in five and eight-week-old *Npc1 ^-/-^* mice (filled columns) relative to *Npc1 ^+/+^* animals (open columns). Data are mean ± SEM, ** *p* < 0.01 *** *p* < 0.001 n=5 per group. unpaired *t* test with Welch’s correction. Data representative of two independent experiments.

In light of decreased serum iron, we measured systemic hepcidin, which has a central role in systemic iron regulation ^28^. Hepcidin levels were increased significantly in male and female *Npc1 ^-/-^* mice at seven-weeks ( *p* < 0.01 and p < 0.005 respectively, n=5) and at nine-weeks-of-age ( *p* < 0.0001 and *p* < 0.05 n=5) ( Figure 3A).

As hepcidin is induced by inflammatory mediators ^1^ we measured hepatic expression of specific pro-inflammatory cytokines. Transcription of TNFα was significantly greater at five and eight weeks-of-age ( *p* < 0.01 n=8). ( Figure 3Bi), IL-1β was not changed ( Figure 3Bii), but IL-1α was significantly elevated in pre–symptomatic and symptomatic *Npc1 ^-/-^* mice ( *p* < 0.005 and *p* < 0.01, n=8), ( Figure 3Biii).

### Splenomegaly and hepatomegaly altered erythropoiesis and disrupted splenic organization in *Npc1 ^-/-^* mice

**Figure 4.**
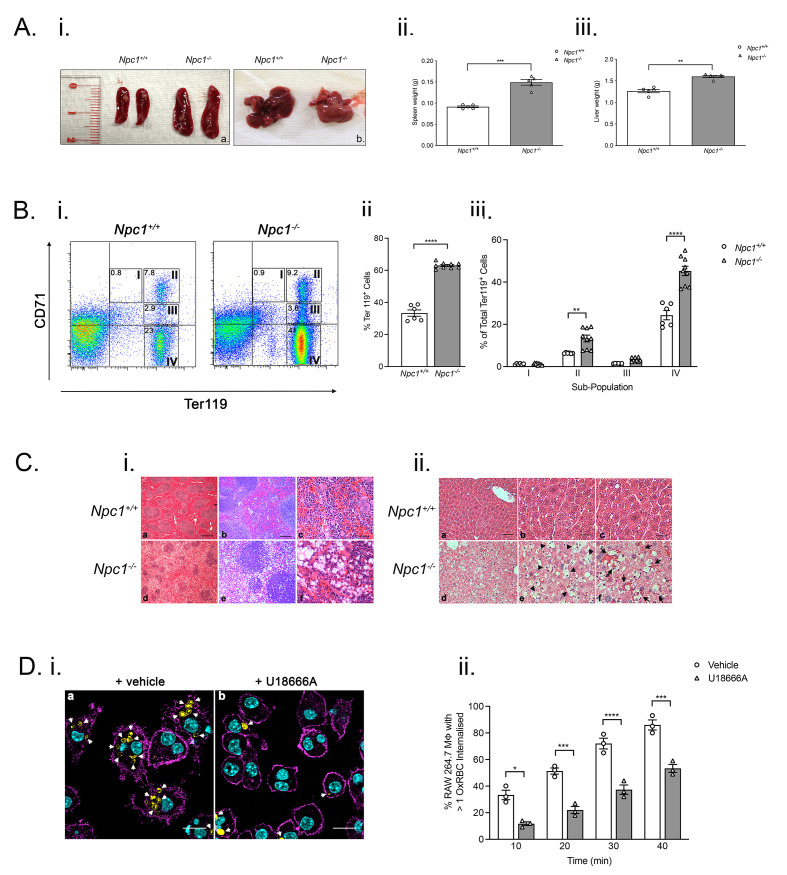
Hepatosplenomegaly, altered erythropoiesis, perturbed splenic architecture and evidence of impaired erythrophagocytosis in *Npc1 ^-/-^* mice. **A**. *Npc1 ^-/-^* mice display increased spleen and liver mass. **(i)**. Representative images of spleen and liver from nine-week-old *Npc1 ^-/-^* and *Npc1 ^+/+^* mice. Histograms of spleen **(ii)** and **(iii)** liver masses from *Npc1 ^+/+^* (open columns) and *Npc1 ^-/-^* animals (filled columns). Data are mean± SEM, ** *p* < 0.01 *** *p* < 0.001 n=5. unpaired *t* test with Welch’s correction. **B**. Perturbed erythropoiesis in *Npc1 ^-/-^* mice. **(i)** Representative FACS profiles of splenic cells from nine-week-old *Npc1 ^+/+^* (left panel) and *Npc1 ^-/-^* mice (right panel) stained with anti-CD71 and anti-Ter119 specific antibodies. Gates indicate position of I, proerythroblasts; II, basophilic; III, polychromatic and IV orthochromatic populations. **(ii)** Quantification of Ter119 ^+^ cell frequencies. Mean± SEM *** *p* < 0.001 n=4–8 per group. unpaired *t* test with Welch’s correction **(iii)** frequencies of sub-populations I-IV. ** *p* < 0.01 **** *p* < 0.0001 n=7–9 per group. unpaired *t* test with Welch’s correction. Data are representative of three independent experiments. **C**. Altered splenic architecture and presence of foamy macrophages and nearby erythrocytes in livers from nine-week-old *Npc1 ^-/-^* mice. **(i)** Representative images of Masson trichrome stained spleen sections from nine-week-old *Npc1 ^+/+^* (panels a-c) and *Npc1 ^-/-^* (panels d-f) mice. Scale bar: panels a and d, 250 μm; b and e, 100 μm; c and f, 25 μm. **(ii)**. Hematoxylin/eosin-stained liver sections from nine-week-old *Npc1 ^+/+^* (panels a–c) and *Npc1 ^-/-^* mice (panels d–f). Arrowheads indicate examples of macrophages with foamy appearance; arrows indicate erythrocytes in close proximity to foamy macrophages. Scale bar; panels a and d, 50 μm; b, c, e, and f, 25 μm. Images were captured with a Zeiss Axioplan 2 microscope using Axiovision 2.0 software. **D**. Impaired *in vitro* phagocytosis of oxidized sheep erythrocytes by U18666A-treated RAW 264.7 macrophages. **(i)** Representative confocal microscopy images of vehicle-treated (left panel) and U18666A-treated RAW 264.7 macrophages (right panel) that have been co-incubated with oxidized sheep red blood cells. Arrows indicate examples of internalized erythrocytes (green). Cyan represents actin staining. Scale bar; 20 μm. Cells were imaged on a Leica TCS SP8 confocal microscope with LAS X software **(ii)**. Frequencies of oxidized sheep erythrocytes internalized by vehicle-treated (open columns) and U18666A-treated (filled columns) RAW 264.7 macrophages. Mean ± SEM, n=minimum of 3 x 100 cells counted for each treatment. Data is representative of three independent experiments. * *p* < 0.05, *** *p* < 0.001, **** *p* < 0.0001. unpaired *t* test with Welch’s correction.

Anaemia affects erythropoiesis ^1^ and we therefore examined the erythrocytic compartment in mutant mice. Nine-week-old *Npc1 ^-/-^* mice exhibited splenomegaly ( *p* < 0.005; n=5) ( Figures 4Ai and ii). Livers from nine-week-old *Npc1 ^-/-^* mice were paler in appearance and had significantly greater wet weight mass ( *p* < 0.01, n=8) ( Figures 4Ai and iii). We analyzed the frequency and maturation of splenic erythroid lineage cells by flow cytometry ( Figure 4Bi). At nine-weeks-of-age, there was a significant increase in Ter119 ^+^ erythroblasts in *Npc1 ^-/-^* mice ( *p* < 0.01, n=8) consistent with enhanced erythropoiesis ( Figure 4Bii). There were significantly more orthochromatic erythroblasts (Ter1119 ^hi^ CD71 ^lo^; Figure 4Bi, Region IV) indicating a higher frequency of nucleated red cells in *Npc1 ^-/-^* mice ( *p* < 0.01 n=7–9) ( Figures 4Bi and iii). However, maturation of splenic pro-erythroblasts into chromatophilic erythroblasts was unaltered ( Figure 4Biii, gates I–III). Furthermore, histochemical staining revealed disorganized splenic architecture. Lymphoid follicles appeared relatively normal albeit with evidence of vacuolated cells. In contrast, red pulp regions were disorganized and contained large numbers of vacuolated cells ( Figure 4Ci).

### Impaired erythrophagocytosis in *Npc1 ^-/-^* mice and a cellular model of NPC1 disease

Aged erythrocytes are cleared by reticuloendothelial macrophages and defective phagocytic clearance has the potential to impact upon iron recycling ^1^. Foamy macrophages were apparent in nine-week-old *Npc1 ^-/-^* liver ( Figure 4Cii, panels e and f) but were absent from controls ( Figure 4Cii, panels b and c). Non-ingested erythrocytes were frequently observed in close proximity to foamy macrophages in *Npc1 ^-/-^* mice ( Figure 4Cii, panel f). In light of these data suggesting defective clearance of senescent RBCs *in vivo,* we used an *in vitro* phagocytosis assay to investigate phagocytosis of red cells. RAW 264.7 murine macrophages treated with U18666A, which inhibits NPC1 ^29^ ingested significantly fewer oxidized sRBC (Ox-sRBC) than vehicle-treated macrophages (10 min, *p* < 0.05; 20 min, *p* < 0.005; 30 min, *p* < 0.0001; 40 min, *p* < 0.005, n=200) ( Figures 4Di and ii), in line with what was observed in *Npc1 ^-/-^* liver. 

### Multiple hematological parameters tend towards the lower end of the normal range in NPC1 patients

**Figure 5.**
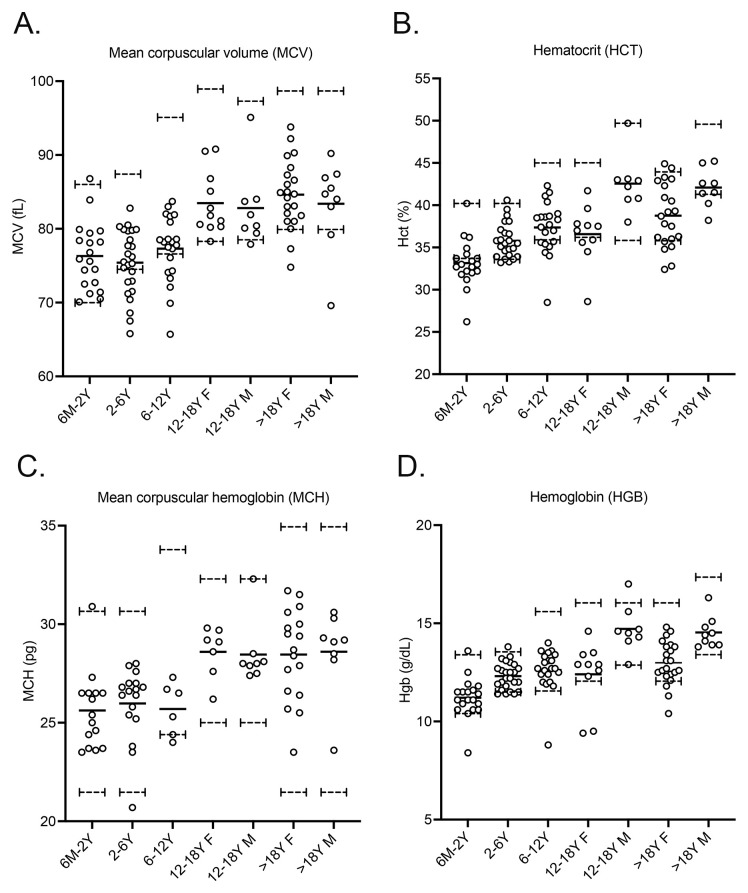
NPC patients have hematological parameters that cluster at the low end of the normal range. Plots of patient values: **A**. Mean corpuscular volume, **B**. hematocrit, **C**. corpuscular hemoglobin and **D**. hemoglobin. Data are mean ± SEM, n=19 (six months – two years); 25 (2–6 years); 21 (6–12 years); 11 (12–18 years females); 8 (12–18 years males); 21 (>18 years females); 9 (> 18 years males). Dashed lines indicate values at the limits of the normal range ^30, 31^.

To ascertain whether evidence of systemic iron dysregulation and hematological abnormalities in *Npc1 ^-/-^* mice were relevant clinically, blood samples from NPC1 patients were analyzed. A total of 114 NPC1 patients were examined of which 54 were male and 60 were female. Fifty-two patients (45%) were taking off-label miglustat (Zavesca). Thirty-eight (33%) were taking a multi-vitamin containing iron. Exclusion of these subjects yielded results similar to when they were included. Although none of the values for MCV, HCT and MCH and HGB in NPC1 patients were statistically different from normal values many were clustered at the lower end of the normal range or were within the lower half ( Figures 5A–D).

### Serum iron, iron saturation, serum ferritin and Tf saturation are significantly different in NPC1 patients

**Figure 6.**
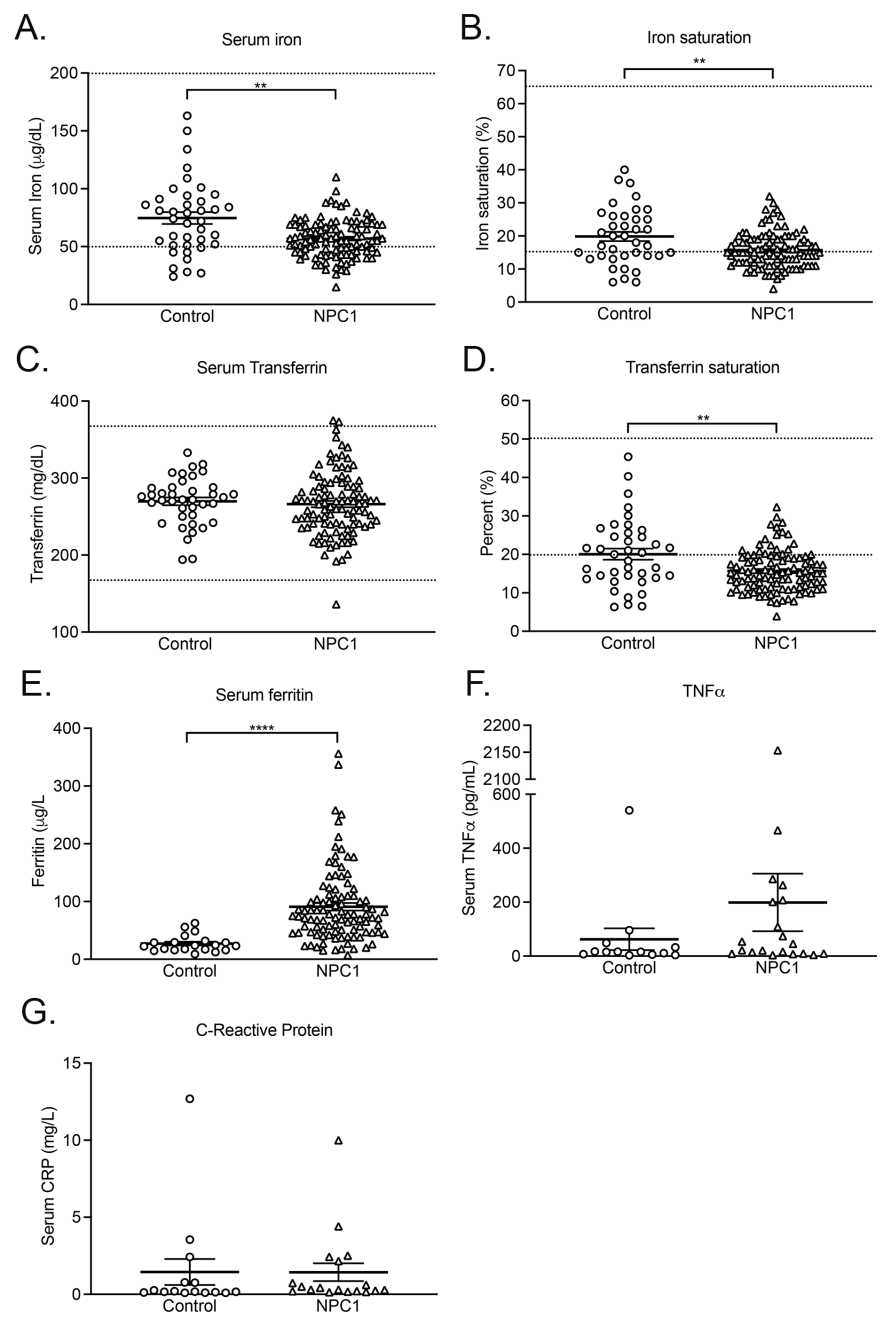
NPC1 patients have significantly lower serum iron, iron and transferrin saturation, but increased ferritin levels. Plots of patient **A**. serum iron, **B**. iron saturation, **C**. serum transferrin, **D**. transferrin saturation, **E**. serum ferritin **F**. systemic TNFα and **G**. systemic C-reactive protein. Mean ± SEM, * *p* < 0.05,** *p* < 0.01 *** *p* < 0.001 unpaired *t* test with Welch’s correction For serum iron, n=105 for NPC1 patients, 39 for controls; iron saturation, n=104 for NPC1, n=38 for controls; serum transferrin, n=104 for NPC1, 39 for controls; transferrin saturation, n=39 for control group, 105 for NPC1 patients; serum ferritin, n=20 for control and 100 for NPC1, serum TNF-α n=11 for control and 20 for NPC1 and for C-reactive protein, n=15 for control group (mean age 12.7 ± 5.8 years), 18 for NPC1 patients (mean age 11.6 ± 7.9 years. Dashed lines indicate values at the limits of the normal range of the various parameters ^30, 31^.

Serum iron was significantly lower in NPC1 patients ( *p* < 0.01, n=105), as was iron saturation ( *p* < 0.05, n=104) and Tf saturation ( *p* < 0.05, n=104) ( Figures 6A, B and D). Serum ferritin was significantly elevated in patients ( *p* < 0.005, n=100) ( Figure 6E). None of these parameters correlated with disease severity (Extended Figure 3). Serum transferrin was not significantly different between the two populations ( Figure 6C). Although serum TNFα in NPC1 patients was slightly elevated, in 6 of the 20 NPC1 patients analyzed, values were not statistically different from age-matched controls ( Figure 6F). TNFα values for two patients and one control were below limit of detection. Plasma CRP was not different between the two groups ( Figure 6G).

### Ferritin and transferrin levels are significantly different in NPC1 patient cerebrospinal fluid (CSF)

**Figure 7.**
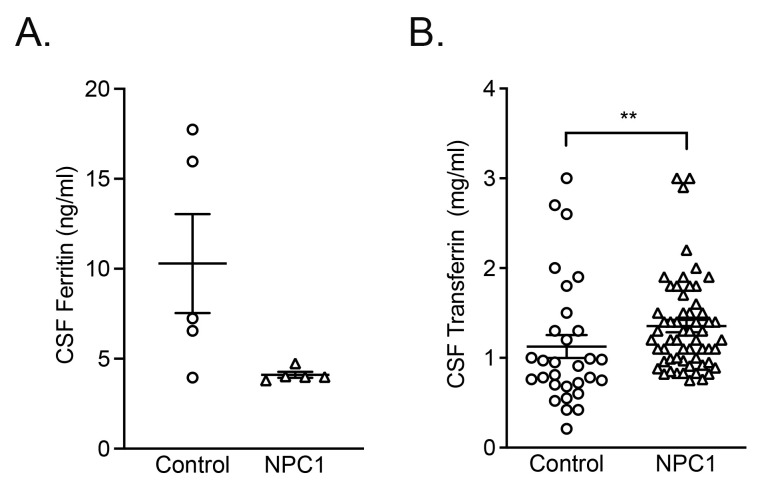
Ferritin deficiency and increased transferrin in the CSF of NPC1 patients. Plots of **A**. CSF ferritin and **B**. CSF transferrin levels in control and NPC1 patients. Mean ± SEM, * *p* < 0.05, ** *p* < 0.01. unpaired *t* test with Welch’s correction. n=5 for ferritin determinations, n=30, n=58 for control and NPC1 patients for transferrin determinations.

We then analyzed CSF to evaluate iron metabolism in the CNS. With the exception of a single individual, all controls had measurable levels of CSF ferritin, whereas all NPC1 patients were below the assay detection threshold ( *p* < 0.05, n=5) ( Figure 7A). Transferrin levels in patient CSF was significantly higher than in controls ( *p* < 0.01, n=58 and n=30 respectively).

## Discussion

Iron homeostasis is achieved through the coordinated activities of multiple proteins that regulate metal ion uptake, storage, efflux, and recycling, in addition to systemic hormonal regulation ^1^. Disruption of lysosome-dependent activities in NPC1 has the potential to affect several of these processes. We therefore explored iron metabolism in NPC1 at multiple levels in an authentic murine model and a large cohort of patients (> 100 individuals) to obtain a broad picture of clinical phenotypes and identify specific abnormalities.

Here, we report multiple hematological changes in *Npc1* ^-/-^ mice that include erythrocyte and hemoglobin-related parameters, systemic iron deficiency, abnormal erythropoiesis and impaired erythrophagocytosis, which confirm disrupted iron homeostasis ^32^. NPC1 patients exhibited milder erythrocytic phenotypes, including low serum iron and Tf saturation and increased serum ferritin ( Table 2). This differential severity is most likely explained by the disparity between residual NPC1 activities in the two species; patients have mutations in *NPC1* that encode partially functional proteins, whereas the mouse model is null for activity. However, genetic diversity may also impact upon the differential disease severity between mice and patients: *Npc1 ^-/-^* mice were all on an identical genetic background (Balb/c) and unidentified modifier genes may have the potential to affect patient phenotypes”. The evidence of impaired iron homeostasis in NPC1 is in agreement with our previous study ^33^ and independent findings reported by Bush and colleagues ^34^ that suggest altered transition metal homeostasis in NPC1 mice and patients. Here, we provide important mechanistic insights and identify several pathophysiological mechanisms that may be responsible for altered systemic iron metabolism in NPC1 disease.

**Table 2.** Hematological pathologies shared between *Npc1 ^-/-^* mice and NPC1 patients.

Parameter	*Npc1 ^-/-^* mouse	Patient
MCV	Decreased ^a^	Lower end of normal range
MCH	Decreased ^a^	Lower end of normal range
HCT	Decreased ^b^	Lower end of normal range
RDW	Increased ^b^	Lower end of normal range
HGB	Decreased ^b^	Lower end of normal range
Microcytic erythrocytes	Yes	Yes or absent ^c,d^
Serum iron	Decreased	Decreased ^e^
sTfrc	Increased	NR
Serum ferritin	Increased	Increased
Liver ferritin	Reduced	Reduced ^c,d^
Hepatosplenamegaly	Yes	Yes or absent ^f^

^a^. pre-symptomatic to late symptomatic mice; ^b^. late symptomatic mice; ^c^. 35; ^d^. 36; ^e^. 34; ^f^. 37. **Abbreviations:** MCV, mean corpuscular volume; MCH, mean corpuscular hemoglobin; HCT, hematocrit; RDW, red cell distribution width; HGB, hemoglobin; sTfrc, soluble transferrin receptor; NR, not reported.

*Npc1* ^-/-^ mouse erythrocytes were microcytic and exhibited significantly decreased MCV and HCT, features that have been documented for iron deficiency anemia, thalassemia and anemia of chronic diseases ^38^. However, we cannot exclude the possibility that altered cholesterol and sphingolipid composition of the plasma membrane is responsible for the structural defects in *Npc1* ^-/-^ erythrocytes. Four pediatric patients evaluated by Christomanou and colleagues ^36^ did not display microcytic hypochromic anemia, but the same authors report anecdotally other NPC1 patients who did show these clinical features ^35^, confirming hematological heterogeneity in this lysosomal storage disorder. Furthermore, a case report of a 29-year-old juvenile-onset patient found mild iron deficiency anaemia ^39^. Although only some NPC1 patient erythrocyte phenotypes were altered, they were less acute than those of the mouse model and tended towards the lower end of the normal range, consistent with possible susceptibility to anemia. Monitoring of reticulocyte cellular indices is the basis for early diagnosis of propensity to develop anemia ^40, 41^.

To characterise further the status of iron homeostasis we measured serum iron levels and expression of hemoglobin-associated molecules. Symptomatic mice and NPC1 patients had significantly reduced serum iron, but in accordance with a less acute phenotype, patient sTfrc was not significantly different from controls, but was significantly higher in *Npc1 ^-/-^* mice. Hung *et al.* ^34^ did not report changes in plasma iron in *Npc1 ^-/-^* mice, but analysed mice appreciably younger (three-weeks and seven-weeks of age) than animals examined here (nine-weeks of age). These authors did however confirm a significant reduction in plasma iron in patients ^34^.

Hepatic expression of L-ferritin was significantly reduced in *Npc1 ^-/-^* mice, as has been described previously in patients ^35, 36^, consistent with functional iron deficiency. Analysis of the relative amount of hepatic iron in *Npc1 ^-/-^* mice did not reveal a consistent relationship; whilst there was a significant decrease at seven weeks, comparable to the findings of Hung *et al.* ^34^, we found no significant difference at later time points. The deficiency in total liver iron in *Npc1 ^-/-^* mice is likely to be less pronounced because of hepatomegaly. It may be that altered distribution of iron within tissues, rather than changes in total levels, results in functional deficiency.

Low serum iron likely results from systemic iron dysregulation and systemic pro-inflammatory responses in NPC1. The peptide hormone hepcidin is a critical regulator of systemic iron via its inhibition of iron adsorption in the duodenum and release from macrophages and hepatocytes ^1^. Hepcidin production is regulated primarily by three mechanisms: an iron-regulated pathway, an inflammatory pathway and erythropoiesis. Hepcidin synthesis is stimulated by high iron stores as well as by specific pro-inflammatory molecules, whereas increased erythropoiesis suppresses its production ^28^. Circulating levels of hepcidin were significantly higher in symptomatic and late-symptomatic *Npc1 ^-/-^* animals, which is in agreement with the up-regulation of specific immune and pro-inflammatory molecules such as IL-1a that occurs during disease progression. IL-6 has been shown to induce the synthesis of hepcidin ^42^. Whilst enhanced secretion of the cytokine by patient derived fibroblasts in culture has been documented ^43^, to our knowledge there are no published data of systemic levels *in vivo*. NPC1 has a unique combination of iron-related characteristics because it shares commonalities with both iron deficiency anemia and inflammatory induced anemia and other LSDs such as GM2 gangliosidosis ( Table 3). This would suggest that inflammation alone is unlikely to cause loss of iron homeostasis in NPC1. Inflammation may be secondary to loss of lysosomal homeostasis and induction of cellular iron deficiency, as in the case of lysosome alkalinisation ^44^. It would be of interest to investigate whether amelioration of specific inflammatory mediators can restore iron homeostasis and normalize hepcidin levels and separately, whether neutralisation of hepcidin activity ^45^ has benefit. The involvement of erythroferrone, an erythroid regulator of hepcidin ^50^ in NPC1 may also be relevant.

**Table 3.** Comparison of iron-related parameters in NPC1 disease with other disorders.

Parameter	Iron deficiency anemia ^a,^	Inflammatory induced anemia ^b^/anemia of chronic disease ^c^	NPC1 ^d^	GM2 Gangliosidosis ^i^
Serum ferritin	Reduced	Increased	Increased ^h^	
Serum iron	Reduced	Reduced	Reduced ^e, h^	Reduced
Transferrin	Increased	Reduced or Unchanged	Unchanged	Reduced
Tf saturation	Reduced	Reduced	Reduced	Reduced
sTfrc	Increased	Reduced or Unchanged	Increased ^h^	Increased
MCV	Reduced	Reduced or Unchanged	Reduced ^h^	
Hemoglobin	Reduced	Reduced	Reduced ^h^	
Liver ferritin	Reduced	Reduced	Reduced ^f, g^	
Microcytic erythrocytes	Present	Present or absent	Present or absent ^f, h^	
Inflammatory cytokines	Unchanged	Increased	Atypical inflammatory profile ^h^	Increased
Serum hepcidin	Reduced	Increased	Increased ^h^	
Erythropoiesis	NA	Reduced	Reduced ^h^	
MCH	Reduced	Unchanged	Reduced ^h^	Unchanged

a. 46; b. 47; c. 48; d. 37; e. 34; f. 36; g. 35; h. Also in mouse, this study; i. 49. **Abbreviations:** MCV, mean corpuscular volume; Tf saturation, transferrin saturation; sTfrc, soluble transferrin receptor; MCH, mean corpuscular hemoglobin.

We report significant splenomegaly and hepatomegaly in *Npc1 ^-/-^* mice. Although it has a variable age of onset, hepatosplenomegaly is a clinical feature of NPC1, particularly in early infantile forms of disease ^37^, which the *Npc1 ^-/-^* mouse model mimics most closely. Splenomegaly also occurs in other lysosomal storage diseases ^51^. There was also evidence of ineffective erythropoiesis, confirmed by a significantly greater frequency of Ter119 ^+^ erythroblasts and accumulation of nucleated orthochromatic erythroblasts. Erythropoiesis, which occurs in the spleen, involves proliferation and differentiation of progenitors through distinct stages to yield nonnucleated reticulocytes ^52^ and nucleated orthochromatic erythroblasts represent the terminal stages after which nuclei are ejected to become reticulocytes that can circulate ^52^. Production of red blood cells has a major demand for iron ^1^ and the disruption of the process is consistent with insufficiency. This phenotype together with evidence of disorganised splenic architecture is characteristic of iron deficiency anaemia ^1^.

Because the majority of iron resides in the erythrocytic compartment and erythrocytes have a relatively short life span, efficient recycling of iron from senescent erythrocytes is critical ^1^. Myeloid cells in the spleen and liver phagocytose aged erythrocytes and iron is recycled in order to meet the demand for erythropoiesis ^16^. In experimental models of anaemia, senescent red cells are ingested by monocytes that accumulate in the liver ^53^. We observed non-ingested RBCs in the liver *in vivo* and impaired phagocytosis *in vitro* consistent with defective removal of aged erythrocytes (and hence decreased recycling of iron) in *Npc1 ^-/-^* mice. Diminished phagocytosis and the potential to impact significantly upon heme/iron recycling within the phago-lysosomal system in NPC1 is currently under investigation.

NPC1 is defined clinically as a progressive neurodegenerative disease and although we could not detect alteration in total levels of iron in the *Npc1 ^-/-^* mouse brain, perhaps because of the normal low levels of the metal ion in this organ, there was reduced CSF ferritin in patients, indicative of altered iron metabolism in the CNS. Hung *et al.* ^34^ reported moderate increases in iron content of *Npc1 ^-/-^* cerebellum and cerebrum, and it may be that our analysis of intact mouse brains obscured regional differences. A probable consequence of iron deficiency in the NPC1 brain is compromised in mitochondrial function and capacity resulting in reduced oxidate energy and further neuronal dysfunction. In light of the evidence of increased cerebellar and cerebral iron, Hung *et al.* ^54^ tested for benefit of iron chelation through injection of deferiprone into NPC1 mice but were unable to measure any improvement in either onset of disease symptoms or lifespan. Loss of brain iron homeostasis is implicated in the pathogenesis of common neurodegenerative disorders, including Parkinson’s and Alzheimer’s diseases ^55^ and may therefore contribute to neurodegeneration in NPC1. Brain iron accumulation has been reported in other rare neurodegenerative diseases, such as pantothenate kinase-associated neurodegeneration, neuroferritinopathy, aceruloplasminemia, Kufor Rakeb syndromes and fatty acid hydroxylase associated neurodegeneration ^55– 57^. The finding of altered levels of iron-related molecules in patient CSF may be relevant for the identification of biomarkers to monitor therapeutic interventions. Investigation of additional CNS iron phenotypes, and distribution both regionally and at the sub-cellular level in mutant mice and patients is therefore merited.

Previously, we identified systemic iron dysregulation-induced haematological changes in murine models of GM1 and GM2 gangliosidoses ^49^. We documented progressive depletion of tissue iron, including in the brain, hematological profiles indicative of iron deficiency and demonstrated that dietary iron supplementation provided functional benefit ^49^. In comparison with GM1/GM2 gangliosidosis mice, *Npc1* ^-/-^ mice exhibited more severe hematological abnormalities, increased circulating hepcidin, abnormal hepatic pro-inflammatory cytokines profiles, erythropoiesis and erythrophagocytosis defects. Hyperferritinemia, iron accumulation and elevated hepcidin are pathologies also described in Gaucher disease ^58^. Disrupted iron metabolism is therefore common to multiple lysosomal storage diseases, emphasising the organelle is critical for iron homeostasis. However, the occurrence of disease-specific phenotypes suggests distinct mechanisms that will require further investigation. Furthermore, lysosomal protease activities and lysosomal acidification are crucial for the degradation of ferritin complexes and utilization of iron ^11, 13^. NPC1 activity might contribute to other lysosomal mechanisms that impact upon iron homeostasis, such as iron incorporation into ferritin subunits and iron export from lysosomes into the cytosol ^11, 59^. Iron is effluxed from the lysosome to maintain cytoplasmic concentrations ^44^. It is pertinent that NPC1 protein can mediate the intracellular transport of copper ^60^ and belongs to the resistance-nodulation-cell division (RND) permease superfamily that in prokaryotes function as proton symporters in the coupled efflux of multiple substrates including metals ^61^. Intriguingly, genetic screens to identify protein binding partners of Nrc1, the yeast orthologue of NPC1 protein, identified the iron transporter Fth1 that is responsible for the movement of intravacuolar iron stores ^62, 63^.

## Conclusions

In conclusion, we have identified significant changes in reticulocyte indices and alterations and iron regulatory proteins in an authentic murine model of NPC1 with some phenotypes in NPC1 patients, albeit milder. This profile includes elements characteristic of both inflammatory and non-inflammatory iron deficiencies, which to our knowledge is unique to NPC1 ( Table 3). Although we were unable to detect a correlation between specific hematological parameters and clinical severity it should be noted that the latter is a scale almost entirely based upon evaluation of neurological symptoms. Importantly, loss of systemic iron homeostasis has the potential to impact upon pathogenesis. Whilst precise details of the mechanisms responsible for iron dysregulation remain to be fully elucidated, our findings have the potential to provide novel insights into the biological functions of the NPC1 protein, identify therapeutic targets and provide peripheral and CNS biomarkers that may be useful for analysis of disease. NPC1 patients may be at risk of systemic iron defects and monitoring of serum iron and blood counts may be important for effective clinical management and the evaluation of therapies. Although the NPC patient data shown here does not support the requirement for iron supplementation, it does highlight that clinicians, particularly those who do not regularly see individuals with NPC should be aware that iron levels may be low, but unless there is evidence of a functional problem there is not an immediate need to supplement.

## Consent

Written informed consent for publication of the patients’ details and their images was obtained from the patients or guardian of the patient.

## Data Availability

Zenodo: Defective iron homeostasis and haematological abnormalities in Niemann-Pick disease type C1. https://doi.org/10.5281/zenodo.6792432 ^27^ This project contains the following underlying data: Data underlying Figure 1: Fig 1 hom f 197.4c001 colour adjusted copy 2.tiff Mouse hematology data.xlsx Fig 1hom m 196.5e003 copy 4.tiff Fig 1wt f 208.3i001 copy 4.tiff Fig 1wt m 196.6c001 copy 4.tiff Data underlying Figure 2: L ferritin spleen WB. L ferritin liver WB Serum sTfrc entire WB Actin liver colour WB b-Actin spleen WB b-Actin liver WB L ferritin & liver WB quantification.xlsx Hepatic iron levels.xlsx. Mouse serum iron.xlsx Q-PCR ferroportin vs actin. xlsx Q-PCR Tfrc rel to HPRT.xls Serum sTfrc WB quantification.xlsx Data underlying Figure 3: Hepcidin levels.xlsx Q-PCR IL-1a vs HPRT.xlsx Q-PCR IL-1b vs HPRT.xlsx Q-PCR TNFa vs_HPRT.xlsx Data underlying Figure 4: WT and NPC1 liver image WT and NPC1 spleen image Frequency of Splenic Ter119 Subpopulations.xlsx oxRBC phagocytosis. Xlsx RAW + U18666A + oxRBC image RAW + vehicle + oxRBC image Spleen FACS files CD71 & Ter 119 (folder) Splenic Ter119 cells.xlsx Npc ^-/-^ liver panel d Npc ^-/-^ panel f Npc ^-/-^ liver area in panel e Npc ^-/-^ Liver area in panel f Npc ^+/+^ Liver area in panel b Npc ^+/+^ liver panel b Npc ^+/+^ Liver panel c Npc ^+/+^ Liver area in panel c Npc ^+/+^ Liver panel a Npc ^+/+^ Liver area in panel b. Npc ^-/-^ Spleen panel d Npc ^+/+^ Spleen panel c Npc ^-/-^ Spleen panel f Npc ^-/-^ Spleen area in panel d Npc ^-/-^ Spleen panel e Npc ^+/+^ Spleen panel b Npc ^+/+^ Spleen region in panel d WT and Npc ^-/-^ Spleen and liver weights. xlsx Data underlying Figure 5: NPC Patient Haematology No names.xls Data underlying Figure 6: Patient serum data.xlsx Data underlying Figure 7: CSF Ferritin and transferrin data.xlsx Zenodo: Defective iron homeostasis and haematological abnormalities in Niemann-Pick disease type C1. https://doi.org/10.5281/zenodo.6792432 ^27^ This project contains the following extended data: Patient Haem vs severity score.xlsx **Extended Figure 1. Erythrocyte number and mean corpuscular hemoglobin concentration (MCHC) are not changed in *Npc1 ^-/-^* mice.** Histograms of RBC number ( **A**) and MCHC ( **B**) in *Npc1 ^+/+^* mice (open columns) and *Npc1 ^-/-^* mice (filled columns) at specified ages. n=5–11 mice per group **Extended Figure 2. Brain iron levels are not significantly changed in *Npc1 ^-/-^* mice.** Graph of iron content of *Npc1 ^+/+^* brain (filled circles) and *Npc1 ^-/-^* brain (filled triangles) at indicated ages. N=3 per group. **Extended Figure 3. Serum iron levels, serum iron saturation, systemic ferritin and serum transferrin levels in NPC1 patients do not correlate with clinical severity**. Plots for serum iron (n=105), serum iron saturation (n=104), serum ferritin (n=100) and serum transferrin (n=104) vs clinical severity as determined by annualized severity increment score (ASIS) ^26^. Zenodo: ARRIVE checklist for ‘Defective iron homeostasis and hematological abnormalities in Niemann-Pick disease type C1’, https://doi.org/10.5281/zenodo.6792432 ^27^ Data are available under the terms of the Creative Commons Attribution 4.0 International license (CC-BY 4.0).

## References

[1] Muckenthaler MU Rivella S Hentze MW : A Red Carpet for Iron Metabolism. *Cell.* 2017;168(3):344–361. 10.1016/j.cell.2016.12.034 28129536 PMC5706455

[2] De Domenico I McVey Ward D Kaplan J : Regulation of iron acquisition and storage: consequences for iron-linked disorders. *Nat Rev Mol Cell Biol.* 2008;9(1):72–81. 10.1038/nrm2295 17987043

[3] Hentze MW Muckenthaler MU Galy B : Two to tango: regulation of Mammalian iron metabolism. *Cell.* 2010;142(1):24–38. 10.1016/j.cell.2010.06.028 20603012

[4] Saftig P Klumperman J : Lysosome biogenesis and lysosomal membrane proteins: trafficking meets function. *Nat Rev Mol Cell Biol.* 2009;10(9):623–635. 10.1038/nrm2745 19672277

[5] Mancias JD Wang X Gygi SP : Quantitative proteomics identifies NCOA4 as the cargo receptor mediating ferritinophagy. *Nature.* 2014;509(7498):105–109. 10.1038/nature13148 24695223 PMC4180099

[6] De Domenico I Ward DM Kaplan J : Hepcidin regulation: ironing out the details. *J Clin Invest.* 2007;117(7):1755–1758. 10.1172/JCI32701 17607352 PMC1904333

[7] Dycke C Charbonnier P Pantopoulos K : A role for lysosomes in the turnover of human iron regulatory protein 2. *Int J Biochem Cell Biol.* 2008;40(12):2826–2832. 10.1016/j.biocel.2008.05.015 18582596

[8] Liu MZ Kong N Zhang GY : The critical role of ferritinophagy in human disease. *Front Pharmacol.* 2022;13:933732. 10.3389/fphar.2022.933732 36160450 PMC9493325

[9] Schiavi A Strappazzon F Ventura N : Mitophagy and iron: two actors sharing the stage in age-associated neuronal pathologies. *Mech Ageing Dev.* 2020;188:111252. 10.1016/j.mad.2020.111252 32330468

[10] de Back DZ Kostova EB van Kraaij M : Of macrophages and red blood cells; a complex love story. *Front Physiol.* 2014;5:9. 10.3389/fphys.2014.00009 24523696 PMC3906564

[11] Asano T Komatsu M Yamaguchi-Iwai Y : Distinct mechanisms of ferritin delivery to lysosomes in iron-depleted and iron-replete cells. *Mol Cell Biol.* 2011;31(10):2040–2052. 10.1128/MCB.01437-10 21444722 PMC3133360

[12] Kidane TZ Sauble E Linder MC : Release of iron from ferritin requires lysosomal activity. *Am J Physiol Cell Physiol.* 2006;291(3):C445–455. 10.1152/ajpcell.00505.2005 16611735

[13] Radisky DC Kaplan J : Iron in cytosolic ferritin can be recycled through lysosomal degradation in human fibroblasts. *Biochem J.* 1998;336(Pt 1):201–205. 10.1042/bj3360201 9806901 PMC1219858

[14] De Domenico I Ward DM Kaplan J : Autophagy, ferritin and iron chelation. *Autophagy.* 2010;6(1):157. 10.4161/auto.6.1.10587 20009528

[15] Richardson DR Lane DJ Becker EM : Mitochondrial iron trafficking and the integration of iron metabolism between the mitochondrion and cytosol. *Proc Natl Acad Sci U S A.* 2010;107(24):10775–10782. 10.1073/pnas.0912925107 20495089 PMC2890738

[16] Soares MP Hamza I : Macrophages and Iron Metabolism. *Immunity.* 2016;44(3):492–504. 10.1016/j.immuni.2016.02.016 26982356 PMC4794998

[17] Vanier MT Millat G : Niemann-Pick disease type C. *Clin Genet.* 2003;64(4):269–281. 10.1034/j.1399-0004.2003.00147.x 12974729

[18] Walkley SU Suzuki K : Consequences of NPC1 and NPC2 loss of function in mammalian neurons. *Biochim Biophys Acta.* 2004;1685(1–3):48–62. 10.1016/j.bbalip.2004.08.011 15465426

[19] Frolov A Zielinski SE Crowley JR : NPC1 and NPC2 regulate cellular cholesterol homeostasis through generation of low density lipoprotein cholesterol-derived oxysterols. *J Biol Chem.* 2003;278(28):25517–25525. 10.1074/jbc.M302588200 12719428

[20] Lloyd-Evans E Morgan AJ He X : Niemann-Pick disease type C1 is a sphingosine storage disease that causes deregulation of lysosomal calcium. *Nat Med.* 2008;14(11):1247–1255. 10.1038/nm.1876 18953351

[21] Lloyd-Evans E Platt FM : Lipids on trial: the search for the offending metabolite in Niemann-Pick type C disease. *Traffic.* 2010;11(4):419–428. 10.1111/j.1600-0854.2010.01032.x 20059748

[22] Höglinger D Burgoyne T Sanchez-Heras E : NPC1 regulates ER contacts with endocytic organelles to mediate cholesterol egress. *Nat Commun.* 2019;10(1):4276. 10.1038/s41467-019-12152-2 31537798 PMC6753064

[23] Loftus SK Morris JA Carstea ED : Murine model of Niemann-Pick C disease: mutation in a cholesterol homeostasis gene. *Science.* 1997;277(5323):232–235. 10.1126/science.277.5323.232 9211850

[24] Lakhal-Littleton S Wolna M Carr CA : Cardiac ferroportin regulates cellular iron homeostasis and is important for cardiac function. *Proc Natl Acad Sci U S A.* 2015;112(10):3164–3169. 10.1073/pnas.1422373112 25713362 PMC4364209

[25] Olsson M Oldenborg PA : CD47 on experimentally senescent murine RBCs inhibits phagocytosis following Fcgamma receptor-mediated but not scavenger receptor-mediated recognition by macrophages. *Blood.* 2008;112(10):4259–4267. 10.1182/blood-2008-03-143008 18779391

[26] Yanjanin NM Vélez JI Gropman A : Linear clinical progression, independent of age of onset, in Niemann-Pick disease, type C. *Am J Med Genet B Neuropsychiatr Genet.* 2010;153B(1):132–140. 10.1002/ajmg.b.30969 19415691 PMC2798912

[27] Chen OCW Siebel S Colaco A : Defective iron homeostasis and haematological abnormalities in Niemann-Pick disease type C1. *Zenodo.* [Dataset].2022. 10.5281/zenodo.6792432 PMC10090865 37065726

[28] Rishi G Wallace DF Subramaniam VN : Hepcidin: regulation of the master iron regulator. *Biosci Rep.* 2015;35(3):e00192. 10.1042/BSR20150014 26182354 PMC4438303

[29] Lu F Liang Q Abi-Mosleh L : Identification of NPC1 as the target of U18666A, an inhibitor of lysosomal cholesterol export and Ebola infection. *eLife.* 2015;4:e12177. 10.7554/eLife.12177 26646182 PMC4718804

[30] Soldin SJ : Pediatric Reference Intervals.(ed 7th): AACC Press;2011. Reference Source

[31] Hughes HK Kahl LK : The Harriet Lane Handbook.(ed 21st): Elsevier;2017. Reference Source

[32] Stein J Hartmann F Dignass AU : Diagnosis and management of iron deficiency anemia in patients with IBD. *Nat Rev Gastroenterol Hepatol.* 2010;7(11):599–610. 10.1038/nrgastro.2010.151 20924367

[33] Argüello G Martinez P Peña J : Hepatic metabolic response to restricted copper intake in a Niemann-Pick C murine model. *Metallomics.* 2014;6(8):1527–1539. 10.1039/c4mt00056k 24901380

[34] Hung YH Faux NG Killilea DW : Altered transition metal homeostasis in Niemann-Pick disease, type C1. *Metallomics.* 2014;6(3):542–553. 10.1039/c3mt00308f 24343124 PMC4178950

[35] Christomanou H Vanier MT Santambrogio P : Deficient ferritin immunoreactivity in tissues from niemann-pick type C patients: extension of findings to fetal tissues, H and L ferritin isoforms, but also one case of the rare Niemann-Pick C2 complementation group. *Mol Genet Metab.* 2000;70(3):196–202. 10.1006/mgme.2000.3004 10924274

[36] Christomanou H Kellermann J Linke RP : Deficient ferritin immunoreactivity in visceral organs from four patients with Niemann-Pick disease type C. *Biochem Mol Med.* 1995;55(2):105–115. 10.1006/bmme.1995.1040 7582867

[37] Vanier MT : Niemann-Pick disease type C. *Orphanet J Rare Dis.* 2010;5:16. 10.1186/1750-1172-5-16 20525256 PMC2902432

[38] Goodnough LT Nemeth E Ganz T : Detection, evaluation, and management of iron-restricted erythropoiesis. *Blood.* 2010;116(23):4754–4761. 10.1182/blood-2010-05-286260 20826717

[39] Lad M Thomas RH Anderson K : Niemann-Pick type C: contemporary diagnosis and treatment of a classical disorder. *Pract Neurol.* 2019;19(5):420–423. 10.1136/practneurol-2019-002236 31243140 PMC6839726

[40] Brugnara C : Reticulocyte cellular indices: a new approach in the diagnosis of anemias and monitoring of erythropoietic function. *Crit Rev Clin Lab Sci.* 2000;37(2):93–130. 10.1080/10408360091174196 10811141

[41] Thomas C Thomas L : Biochemical markers and hematologic indices in the diagnosis of functional iron deficiency. *Clin Chem.* 2002;48(7):1066–1076. 10.1093/clinchem/48.7.1066 12089176

[42] Nemeth E Rivera S Gabayan V : IL-6 mediates hypoferremia of inflammation by inducing the synthesis of the iron regulatory hormone hepcidin. *J Clin Invest.* 2004;113(9):1271–1276. 10.1172/JCI20945 15124018 PMC398432

[43] Suzuki M Sugimoto Y Ohsaki Y : Endosomal accumulation of Toll-like receptor 4 causes constitutive secretion of cytokines and activation of signal transducers and activators of transcription in Niemann-Pick disease type C (NPC) fibroblasts: a potential basis for glial cell activation in the NPC brain. *J Neurosci.* 2007;27(8):1879–1891. 10.1523/JNEUROSCI.5282-06.2007 17314284 PMC6673540

[44] Yambire KF Rostosky C Watanabe T : Impaired lysosomal acidification triggers iron deficiency and inflammation *in vivo*. *eLife.* 2019;8:e51031. 10.7554/eLife.51031 31793879 PMC6917501

[45] Sasu BJ Cooke KS Arvedson TL : Antihepcidin antibody treatment modulates iron metabolism and is effective in a mouse model of inflammation-induced anemia. *Blood.* 2010;115(17):3616–3624. 10.1182/blood-2009-09-245977 20053755

[46] Cappellini MD Musallam KM Taher AT : Iron deficiency anaemia revisited. *J Intern Med.* 2020;287(2):153–170. 10.1111/joim.13004 31665543

[47] Ganz T : Anemia of Inflammation. *N Engl J Med.* 2019;381(12):1148–1157. 10.1056/NEJMra1804281 31532961

[48] Madu AJ Ughasoro MD : Anaemia of Chronic Disease: An In-Depth Review. *Med Princ Pract.* 2017;26(1):1–9. 10.1159/000452104 27756061 PMC5588399

[49] Jeyakumar M Williams I Smith D : Critical role of iron in the pathogenesis of the murine gangliosidoses. *Neurobiol Dis.* 2009;34(3):406–416. 10.1016/j.nbd.2009.01.015 19449457

[50] Coffey R Ganz T : Erythroferrone: An Erythroid Regulator of Hepcidin and Iron Metabolism. *Hemasphere.* 2018;2(2):e35. 10.1097/HS9.0000000000000035 31723763 PMC6745900

[51] Platt FM d'Azzo A Davidson BL : Lysosomal storage diseases. *Nat Rev Dis Primers.* 2018;4(1):27. 10.1038/s41572-018-0025-4 30275469

[52] Chen K Liu J Heck S : Resolving the distinct stages in erythroid differentiation based on dynamic changes in membrane protein expression during erythropoiesis. *Proc Natl Acad Sci U S A.* 2009;106(41):17413–17418. 10.1073/pnas.0909296106 19805084 PMC2762680

[53] Theurl I Hilgendorf I Nairz M : On-demand erythrocyte disposal and iron recycling requires transient macrophages in the liver. *Nat Med.* 2016;22(8):945–951. 10.1038/nm.4146 27428900 PMC4957133

[54] Hung YH Lotan A Yeshurun S : Iron chelation by deferiprone does not rescue the Niemann-Pick Disease Type C1 mouse model. *Biometals.* 2020;33(2–3):87–95. 10.1007/s10534-020-00233-5 32100150

[55] Zecca L Youdim MB Riederer P : Iron, brain ageing and neurodegenerative disorders. *Nat Rev Neurosci.* 2004;5(11):863–873. 10.1038/nrn1537 15496864

[56] Oshiro S Morioka MS Kikuchi M : Dysregulation of iron metabolism in Alzheimer's disease, Parkinson's disease, and amyotrophic lateral sclerosis. *Adv Pharmacol Sci.* 2011;2011:378278. 10.1155/2011/378278 22013437 PMC3195304

[57] Schneider SA Bhatia KP : Excess iron harms the brain: the syndromes of neurodegeneration with brain iron accumulation (NBIA). *J Neural Transm (Vienna).* 2013;120(4):695–703. 10.1007/s00702-012-0922-8 23212724

[58] Regenboog M van Kuilenburg AB Verheij J : Hyperferritinemia and iron metabolism in Gaucher disease: Potential pathophysiological implications. *Blood Rev.* 2016;30(6):431–437. 10.1016/j.blre.2016.05.003 27265538

[59] Cheng X Shen D Samie M : Mucolipins: Intracellular TRPML1-3 channels. *FEBS Lett.* 2010;584(10):2013–2021. 10.1016/j.febslet.2009.12.056 20074572 PMC2866799

[60] Yanagimoto C Harada M Kumemura H : Niemann-Pick C1 protein transports copper to the secretory compartment from late endosomes where ATP7B resides. *Exp Cell Res.* 2009;315(2):119–126. 10.1016/j.yexcr.2008.10.022 19007772

[61] Scott C Ioannou YA : The NPC1 protein: structure implies function. *Biochim Biophys Acta.* 2004;1685(1–3):8–13. 10.1016/j.bbalip.2004.08.006 15465421

[62] Colaco A Fernández-Suárez ME Shepherd D : Unbiased yeast screens identify cellular pathways affected in Niemann-Pick disease type C. *Life Sci Alliance.* 2020;3(7):e201800253. 10.26508/lsa.201800253 32487688 PMC7283134

[63] Urbanowski JL Piper RC : The iron transporter Fth1p forms a complex with the Fet5 iron oxidase and resides on the vacuolar membrane. *J Biol Chem.* 1999;274(53):38061–38070. 10.1074/jbc.274.53.38061 10608875

