# Supplementary material for: Defective iron homeostasis and hematological abnormalities in Niemann-Pick disease type C1
Source: Wellcome Open Res. 2023 Apr 3;7:267. Originally published 2022 Oct 20. [Version 2] doi: 10.12688/wellcomeopenres.17261.2 (PMC10090865; doi:10.12688/wellcomeopenres.17261.2)
Supplement: Peer review history

# Peer review: Reviewer response for version 2

**DOI:** 10.21956/wellcomeopenres.21047.r56002

**Published:** 2023-04-11

**Authors:** Caroline Hastings

**Affiliations:** 1 Department of Pediatrics, Division of Hematology Oncology, Benioff Children's Hospital Oakland, University of California San Francisco, Oakland, CA, USA; 2 UCSF Benioff Children's Hospital Oakland, Oakland, CA, USA

I have no further comments and approve this version for indexing.

Is the work clearly and accurately presented and does it cite the current literature?

Yes

If applicable, is the statistical analysis and its interpretation appropriate?

Yes

Are all the source data underlying the results available to ensure full reproducibility?

Yes

Is the study design appropriate and is the work technically sound?

Yes

Are the conclusions drawn adequately supported by the results?

Yes

Are sufficient details of methods and analysis provided to allow replication by others?

Yes

Reviewer Expertise:

NPC

I confirm that I have read this submission and believe that I have an appropriate level of expertise to confirm that it is of an acceptable scientific standard.

# Peer review: Reviewer response for version 2

**DOI:** 10.21956/wellcomeopenres.21047.r56003

**Published:** 2023-04-03

**Authors:** Frank W. Pfrieger

**Affiliations:** 1 Centre National de la Recherche Scientifique, Institute of Cellular and Integrative Neurosciences, Universite de Strasbourg, Strasbourg, Grand Est, France

I approve the article.

Is the work clearly and accurately presented and does it cite the current literature?

Partly

If applicable, is the statistical analysis and its interpretation appropriate?

Yes

Are all the source data underlying the results available to ensure full reproducibility?

Yes

Is the study design appropriate and is the work technically sound?

Yes

Are the conclusions drawn adequately supported by the results?

Yes

Are sufficient details of methods and analysis provided to allow replication by others?

Yes

Reviewer Expertise:

Neuroscience

I confirm that I have read this submission and believe that I have an appropriate level of expertise to confirm that it is of an acceptable scientific standard.

# Peer review: Reviewer response for version 1

**DOI:** 10.21956/wellcomeopenres.19078.r52983

**Published:** 2022-11-08

**Authors:** Caroline Hastings

**Affiliations:** 1 Department of Pediatrics, Division of Hematology Oncology, Benioff Children's Hospital Oakland, University of California San Francisco, Oakland, CA, USA; 2 UCSF Benioff Children's Hospital Oakland, Oakland, CA, USA

This is an interesting idea and well written manuscript. The authors do state there is not an absolute correlation between the mice and humans and give some explanations as to why this is likely so. I think this needs further expansion including the lack of genetic variability in the mice, the early ages of the mice, and likely that NPC mice may have more liver disease than most human patients, which significantly impacts hematopoiesis.

 There is a paucity of human data shown. Though the methods state there were 114 patients in the data base, it does not appear there were that many patients on the scatter plots, so this is confusing. As well, I assume this is data form one time point for the patients? This is not clear (whereas there were several time points for the mice as they aged). We don't know how these values in humans compared for those based on age, disease progression, presence or absence of overt liver disease, or presence of co-morbidities. Iron homestasis is sensitive to all these factors. Additionally, the authors note the measure hematologic parameters in the NPC1 patients were at the lower end of the normal range, a range in which we may expect to see patients with a chronic disease that results in inflammation. Additionally, many patients have nutritional concerns due to dysphagia, weight loss, need for supplementation, etc. which may impact these values. It is noted that the values do not correlate with the clinical severity of NPC. Taken together it seems that that in humans it does not appear the changes noted in iron homestasis are clinically relevant.

 In the conclusion it is stated that monitoring of blood counts and iron status may be important for clinical management and the evaluation of therapies, but it does not seem the findings support this statement. Also, in chronic inflammation it is not clear iron replacement is needed or helpful. The clinical care of these patients requires extensive supportive care to ensure optimal quality of life, and (based on this data as well as general clinical experience with these patients) it does not seem that monitoring for anemia per se due to NPC1 is necessary. It is likely these patients have periodic blood counts for general safety monitoring (and have known thrombocytopenia). Therefore this appears to be too sweeping of a conclusion. It seems more investigation may be necessary in humans, more than can be ascertained from natural history data.

 The CSF iron is interesting and decreased levels in the CSF may be expected in patients with neurodegenerative disorders. It might be interesting to determine if this could be a biomarker in the future with interventional therapies. Taken alone, its clinical relevance it not necessarily any different from an elevated Tau for example. It might be good to add some more thought on the potential utility of this finding (knowing the data is limited due to only 1 time point for the patients).

Is the work clearly and accurately presented and does it cite the current literature?

Yes

If applicable, is the statistical analysis and its interpretation appropriate?

Yes

Are all the source data underlying the results available to ensure full reproducibility?

Yes

Is the study design appropriate and is the work technically sound?

Yes

Are the conclusions drawn adequately supported by the results?

Yes

Are sufficient details of methods and analysis provided to allow replication by others?

Yes

Reviewer Expertise:

NPC

I confirm that I have read this submission and believe that I have an appropriate level of expertise to confirm that it is of an acceptable scientific standard, however I have significant reservations, as outlined above.

# Response

**Published:** 2023-03-17

**Authors:** Fran Platt

**Affiliations:** University of Oxford, UK

Caroline Hastings

 We thank the two reviewers for their constructive comments on our manuscript. We have sought to address each point that has been raised.

 This is an interesting idea and well written manuscript. The authors do state there is not an absolute correlation between the mice and humans and give some explanations as to why this is likely so. I think this needs further expansion including the lack of genetic variability in the mice, the early ages of the mice, and likely that NPC mice may have more liver disease than most human patients, which significantly impacts hematopoiesis. We agree that genetic background and lack of variability in *Npc1 ^-/-^* mice may also influence disease presentation and have included a statement to this effect. “However, genetic diversity may also impact upon the differential disease severity between mice and patients: *Npc1 ^-/-^* mice were all on an identical genetic background (Balb/c) and unidentified modifier genes may affect patient phenotypes”.

 There is a paucity of human data shown. Though the methods state there were 114 patients in the data base, it does not appear there were that many patients on the scatter plots, so this is confusing. As well, I assume this is data form one time point for the patients? This is not clear (whereas there were several time points for the mice as they aged). We don't know how these values in humans compared for those based on age, disease progression, presence or absence of overt liver disease, or presence of co-morbidities. Iron homestasis is sensitive to all these factors. Additionally, the authors note the measure hematologic parameters in the NPC1 patients were at the lower end of the normal range, a range in which we may expect to see patients with a chronic disease that results in inflammation. Additionally, many patients have nutritional concerns due to dysphagia, weight loss, need for supplementation, etc. which may impact these values. It is noted that the values do not correlate with the clinical severity of NPC. Taken together it seems that that in humans it does not appear the changes noted in iron homestasis are clinically relevant.

 We have clarified that 114 NPC patients enrolled in a natural history study are reported and numbers for each specific analysis indicated. We have confirmed that data from only one sampling time point was obtained for each individual participant. Data indicating whether patients were receiving additional dietary supplements etc. is provided within underlying data files that have been uploaded to Zenodo.

 In the conclusion it is stated that monitoring of blood counts and iron status may be important for clinical management and the evaluation of therapies, but it does not seem the findings support this statement. Also, in chronic inflammation it is not clear iron replacement is needed or helpful. The clinical care of these patients requires extensive supportive care to ensure optimal quality of life, and (based on this data as well as general clinical experience with these patients) it does not seem that monitoring for anemia per se due to NPC1 is necessary. It is likely these patients have periodic blood counts for general safety monitoring (and have known thrombocytopenia). Therefore this appears to be too sweeping of a conclusion. It seems more investigation may be necessary in humans, more than can be ascertained from natural history data. We are in agreement that NPC patients require extensive and broad clinical care that extends beyond management of neurological disease. In particular, informing clinicians who may not have extensive experience of caring for affected individuals of the potential for haematological/iron changes may be considered important. We have included a statement to this effect.” Although the NPC patient data shown here does not support the requirement for iron supplementation, it does highlight that clinicians, particularly those who do not regularly see individuals with NPC should be aware that iron levels may be low, but unless there is evidence of a functional problem there is not an immediate need to supplement”.

 The CSF iron is interesting and decreased levels in the CSF may be expected in patients with neurodegenerative disorders. It might be interesting to determine if this could be a biomarker in the future with interventional therapies. Taken alone, its clinical relevance it not necessarily any different from an elevated Tau for example. It might be good to add some more thought on the potential utility of this finding (knowing the data is limited due to only 1 time point for the patients).

 The authors thank the reviewer for this suggestion and will consider whether it can be included in future clinical evaluation studies. We have included a statement to this effect. “The finding of altered levels of iron-related molecules in patient CSF may be relevant for the identification of biomarkers to monitor therapeutic interventions”.

# Peer review: Reviewer response for version 1

**DOI:** 10.21956/wellcomeopenres.19078.r52980

**Published:** 2022-11-07

**Authors:** Frank W. Pfrieger

**Affiliations:** 1 Centre National de la Recherche Scientifique, Institute of Cellular and Integrative Neurosciences, Universite de Strasbourg, Strasbourg, Grand Est, France

The article by Chen and colleagues follows up on previous studies reporting defects in iron homeostasis in patients and in mouse models of Niemann-Pick type C (NPC) disease. The present work adds new results from NPC1 mice and a large cohort of NPC patients and therefore corroborates the more general notion of perturbed transition metal homeostasis due to defects in NPC1. Whether these changes contribute to disease progress remains to be proven. The study is well-designed and comprehensive, experimental approaches are explained in a satisfactory manner, the data are clearly presented. The authors may consider a few points to revise their paper.

- Pg. 3, para 3. The authors explain in their Introduction the importance of lysosomal function for iron homeostasis and they mention some key processes such as ferritinophagy, mitophagy and erythrophagocytosis all requiring intact lysosomes. However, for the non-expert reader, the authors should mention the cell types that perform these tasks. A corresponding statement on pg. 10, para 2 could be moved to the Introduction.
- Figs. 1A,B; 2A,C,D; 3B; 4A,B,D: The authors should remove the black fill from columns as individual data points are barely visible on this background. They should indicate the genotype on the x axis rather than in the legend.
- Pg. 6, Fig. 1A, B; legend: The panels do not show "histograms". Therefore, this term should be replaced.
- Pg. 6, Fig. 1C; legend: The authors may consider to write explicitly what exactly is altered in erythrocytes from mutant mice.
- Pg. 11, Fig. 5, legend: It is unclear what "+/- SEM" relates to as the graphs do not show whiskers.
- Pg. 14, para 1: The authors discuss how inflammatory signals may affect hepcidin expression. Previous studies (e.g. Nemeth *et al.*, 2004 ^2^) showed that IL-6 rather than IL-1 or TNFa enhances the levels of the hormone. Is there evidence that IL-6 levels are changed in NPC mice or patients? The authors may consider to include this point in their discussion.
- Discussion: The authors should consider to cite Hung *et al.* 2020 ^2^ who report the results of iron chelation.
- Discussion, Table 3: It may be interesting to include in the table (and the discussion) another lysosomal disorder and to discuss whether lysosomal defects in general affect iron homeostasis.

Is the work clearly and accurately presented and does it cite the current literature?

Partly

If applicable, is the statistical analysis and its interpretation appropriate?

Yes

Are all the source data underlying the results available to ensure full reproducibility?

Yes

Is the study design appropriate and is the work technically sound?

Yes

Are the conclusions drawn adequately supported by the results?

Yes

Are sufficient details of methods and analysis provided to allow replication by others?

Yes

Reviewer Expertise:

Neuroscience

I confirm that I have read this submission and believe that I have an appropriate level of expertise to confirm that it is of an acceptable scientific standard.

## References

[1] : IL-6 mediates hypoferremia of inflammation by inducing the synthesis of the iron regulatory hormone hepcidin. *J Clin Invest* .2004;113(9) : 10.1172/JCI20945 1271-6 10.1172/JCI20945 15124018PMC398432

[2] : Iron chelation by deferiprone does not rescue the Niemann-Pick Disease Type C1 mouse model. *Biometals* .33(2-3) : 10.1007/s10534-020-00233-5 87-95 10.1007/s10534-020-00233-5 32100150

# Response

**Published:** 2023-03-17

**Authors:** Fran Platt

**Affiliations:** University of Oxford, UK

We thank the two reviewers for their constructive comments on our manuscript. We have sought to address each point that has been raised.

 Frank Pfrieger

 The article by Chen and colleagues follows up on previous studies reporting defects in iron homeostasis in patients and in mouse models of Niemann-Pick type C (NPC) disease. The present work adds new results from NPC1 mice and a large cohort of NPC patients and therefore corroborates the more general notion of perturbed transition metal homeostasis due to defects in NPC1. Whether these changes contribute to disease progress remains to be proven. The study is well-designed and comprehensive, experimental approaches are explained in a satisfactory manner, the data are clearly presented. The authors may consider a few points to revise their paper.

 Pg. 3, para 3. The authors explain in their Introduction the importance of lysosomal function for iron homeostasis and they mention some key processes such as ferritinophagy, mitophagy and erythrophagocytosis all requiring intact lysosomes. However, for the non-expert reader, the authors should mention the cell types that perform these tasks. A corresponding statement on pg. 10, para 2 could be moved to the Introduction. As suggested by the reviewer we have modified the text to provide more information about the processes of ferritinophagy and mitophagy to the non-expert reader and indicated relevant references.

 Figs. 1A,B; 2A,C,D; 3B; 4A,B,D: The authors should remove the black fill from columns as individual data points are barely visible on this background. They should indicate the genotype on the x axis rather than in the legend.

 As requested by this reviewer we have changed the black fill from columns to a paler shading and have increased the size of individual data points to enhance the clarity of the figures (for both within the main paper and extended data figures). The genotypes are indicated for each panel as the format of the individual data points.

- Pg. 6, Fig. 1A, B; legend: The panels do not show "histograms". Therefore, this term should be replaced. We have changed the term “histograms” to “bar charts”.
- Pg. 6, Fig. 1C; legend: The authors may consider to write explicitly what exactly is altered in erythrocytes from mutant mice. We have included the description of “microcytic and irregularly shaped”
- Pg. 11, Fig. 5, legend: It is unclear what "+/- SEM" relates to as the graphs do not show whiskers. We have changed this to “individual data points are shown, together with mean”.
- Pg. 14, para 1: The authors discuss how inflammatory signals may affect hepcidin expression. Previous studies (e.g. Nemeth *et al.*, 2004 ^2^ ) showed that IL-6 rather than IL-1 or TNFa enhances the levels of the hormone. Is there evidence that IL-6 levels are changed in NPC mice or patients? The authors may consider to include this point in their discussion. We have included the statement “IL-6 has been shown to induce the synthesis of hepcidin. Whilst enhanced secretion of the cytokine by patient derived fibroblasts in culture has been documented, to our knowledge there are no published data of systemic levels *in vivo”*. 
- Discussion: The authors should consider to cite Hung *et al.*2020 ^2^ who report the results of iron chelation. We have added “In light of the evidence of increased cerebellar and cerebral iron Hung *et al* tested for benefit of iron chelation through injection of deferiprone into NPC1 mice but were unable to measure any improvement in either onset of disease symptoms or lifespan.”
- Discussion, Table 3: It may be interesting to include in the table (and the discussion) another lysosomal disorder and to discuss whether lysosomal defects in general affect iron homeostasis. The discussion includes the following statements with relation to iron homeostasis in other LSDs. “Previously, we identified systemic iron dysregulation-induced haematological changes in murine models of the LSDs GM1 and GM2 gangliosidoses. We documented progressive depletion of tissue iron, including in the brain, hematological profiles indicative of iron deficiency and demonstrated that dietary iron supplementation provided functional benefit. Disrupted iron metabolism is therefore common to multiple lysosomal storage diseases, emphasising the organelle is critical for iron homeostasis. Haematological parameters for murine GM2 gangliosidosis have been included in Table 3 for comparison.
